# Unveiling the power of TiO_2_ doped ZnO nanomaterial as an effective antimicrobial solution in the leather industry

**DOI:** 10.1016/j.heliyon.2024.e38414

**Published:** 2024-09-29

**Authors:** Asma Irshad, Rabbia Jawad, Uzair Ishtiaq, Nicolas Joly, Bochra Bejaoui, Naceur M'Hamdi, Patrick Martin, Firdous Mubashar

**Affiliations:** a School of Biochemistry and Biotechnology, University of the Punjab, Lahore, Pakistan; b School of Biological Sciences, University of the Punjab, Lahore, Pakistan; c Department of Research and Development, Paktex Industries, 2.5 KM Tatlay Road, Kamoke, Gujranwala, 52470, Pakistan; d Department of Life Sciences, University of Management and Technology, Lahore, Pakistan; e Unite Transformations & Agroresources-ULR7519, Univ. Artois, Unilasalle, F-62408, Bethune, France; f University of Carthage, National Institute of Research and Pysico-chemical analysis (INRAP), Laboratory of Useful Materials, Technopark of Sidi Thabet, Ariana, 2020, Tunisia; g University of Carthage, Faculty of Sciences of Bizerte, Department of Chemistry, 7021, Zarzouna, Bizerte, Tunisia; h University of Carthage, National Agronomic Institute of Tunisia, Research Laboratory of Ecosystems & Aquatic Resources, Tunisia

**Keywords:** Antimicrobial potential, Leather materials, Microbial degradation, Surface protection, TiO_2_ doped ZnO nanoparticles

## Abstract

The surface protection of leather supplies is a major concern worldwide due to its susceptibility to microbial growth. Different methods are employed to protect leather, their results ends up with the environmental pollution and human safety issues. Nanoparticles with excellent antimicrobial potential can provide sustainable protection to leather accessories. The present work represented a comprehensive investigation into the preparation and characterization of titanium dioxide-doped zinc oxide (ZnO/TiO_2_ NPs) nanoparticles and their exploring as a potential antimicrobial agent in the leather industry. ZnO nanoparticles were synthesized through Sol-gel method by the reduction of zinc acetate dihydrate *via* black cardamom seed's extract and subsequently doped with TiO_2_. The optical, structural, and morphological features of nanoparticles were thoroughly scrutinized through UV–visible spectroscopy, XRD, FT-IR, and SEM-EDAX. The UV–visible spectrum showed enhanced performance between 300 and 350 nm and various peaks of the FT-IR spectrum, i.e. 3315.53, 1566.20, 1402.25, 1340.53, 1014.56, 921.97, 690.52, and 677.01 cm^−1^, revealed chemical bonds that prove the correct doping of TiO_2_ in ZnO nanoparticles. The characteristic peaks obtained from XRD at 2Ө of 32°, 35.5°, 37.2°, 47.9°, 55.6° 63.51°, and 70° intimated to the crystal planes of (100), (002), (101), (102), (110), (103), and (112), respectively. SEM-EDAX images revealed the roughly spherical but agglomerated structure of nanoparticles with size 45.44 nm. Furthermore, minimum inhibitory concentration (MIC), antimicrobial potential, and anti-biofilm potential analyses of nanoparticles, against all selected microorganisms (*Aspergillus niger, Staphylococcus aureus,* and *Escherichia coli*) provided valuable insights into physical and biological properties of the nanoparticles. The clear zones of inhibition (29–30 mm) against these pathogenic strains showed exceptional antimicrobial action of the ZnO/TiO_2_ NPs. Overall, these results provide an approachable method to synthesize ZnO/TiO_2_ nanoparticles and their antimicrobial ability will prove to be beneficial for the protection of leather materials from various microbial contaminations.

## Graphical abstract

**Figure:**
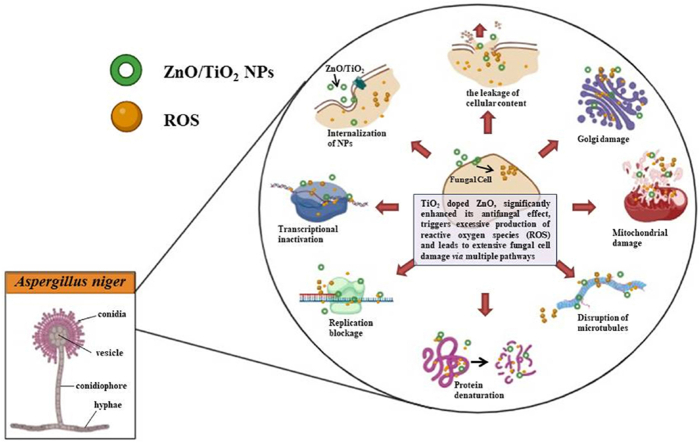

## 1 Introduction

**Fig. 1:**
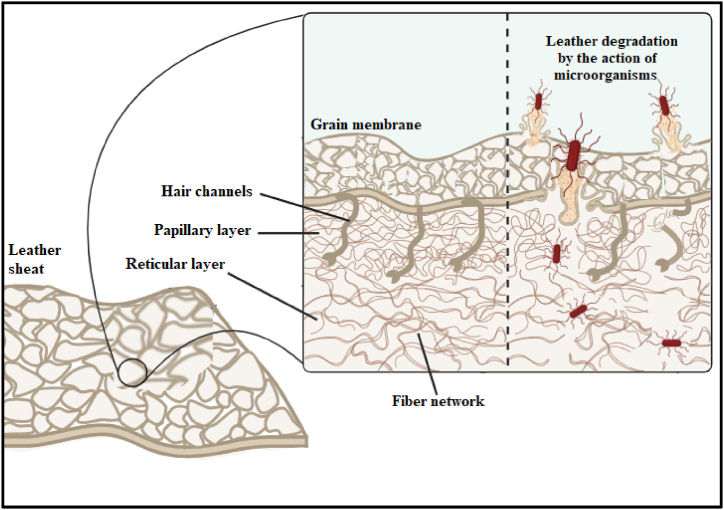
Schamatic diagram shows the mechanism of action imposed by microorganisms to degrade leather suface for their growth and survival.

Today, leather is being used in making many accessories i.e., jackets, gloves, bags, wallets, car seats, etc. Its popularity is due to its exceptional mechanical properties like tensile and shear nature, thickness, surface properties, compression, and weight [1]. All these characteristics make it able to become an essential part of daily life. However, leather is an organic material that contains numerous nutrients used by microorganisms for growth and survival. It is reported that many fungal strains i.e. *Aspergillus niger, A. terreus, A. flavus, Penicillium rubrum, P. notatum, P. oxalicum* [2] and bacterial strains *Staphylococcus aureus, E. coli, Bacillus subtilis* etc can grow on the surface of leather. These pathogenic microorganisms use leather polysaccharides resulting in the breakage of fiber networks and cross linkages of leather materials as shown in Fig. 1 [3]. These chemical properties of leather, together with environmental factors, are key parameters in leather's susceptibility to microorganisms. As a result, the frequency of use of natural leather is increasingly declining compared to treated and artificial leathers [3]. Various features of leather and leather products are frequently compromised due to microbial damage. Microorganisms colonize on the surface of leather products and can cause damage by various means i.e. unpleasant odor, bio deterioration, discoloring, structural damage, healthy safety concerns, storage and transport etc. Leather is primarily comprised of collagen fibers which enhance the flexibility and durability of leather. Bacteria and fungi especially those which have collagen degrading enzymes, weakens the tensile strength of leather leads to make it more susceptible of tearing or other physical destruction [4]. When industries fail to synthesize better quality leather products, it develops severe economic loss to the country and industry itself. Moreover, leather susceptibility to microbial growth increase the demand of advanced storage and transport methods which can safely transport the leather products to the final consumer without damaging. It increases the cost and reduces the purchase of leather products [5].

To prevent the deterioration of leather, manufacturers have resorted to the process of tanning leather materials to make them rot-proof and resistant. However, strict restrictions and requirements are beginning to be imposed worldwide on this technology, due to its serious effects on the environment (China et al., 2020). Leather tanneries generate wastewater effluent and hazardous solid waste, create serious air pollutants and even expose workers to highly harmful chemicals. Recently, many researchers have been looking for alternatives to replace or improve this traditional method. One such alternative technology is the use of nanomaterials as potential antimicrobial agents in the leather industry. Nanoparticles as nanomaterials are increasingly attracting considerable attention in a variety of fields thanks to their diverse multi-functionality and biocompatibility, and their antioxidant, anti-inflammatory and antimicrobial properties [6]. This excellent antimicrobial potential of nanoparticles can help prevent bio deterioration of the leather surface (Elsayed et al., 2021).

Rare earth metals, transition metals, silver nitrate, zinc acetate, zinc sulfate, etc are generally used to prepare nanoparticles with excellent antimicrobial activities against a broad range of microorganisms [7]. There are various methods for synthesizing nanoparticles. The most environmentally friendly is the preparation of nanoparticles from plant extracts [8]. Plant extracts are a rich source of bioactive compounds, notably phenols and flavonoids, and act as antioxidants and reducing agents for green nanoparticle synthesis. A number of projects have been carried out in this area, such as the synthesis of silver nanoparticles for the protection of cotton fabric which was performed by using extracts of some food waste material i.e. pomegranate peel, green tea leaves and avocado seeds. These extracts were considered as good reducing agent for silver nanoparticles [9]. Black cardamom, whose scientific name is *Amomum subulatum*, is also a rich source of secondary metabolites that can help the reduce nanoparticles in an environmentally friendly way, especially as this plant material is available, economical and non-toxic [10]. Zinc oxide (ZnO) nanoparticles prepared from zinc acetate or nitrate, have enhanced antimicrobial potential but they have limited stability in environment [11]. Titanium, cobalt, chromium, manganese, and iron are transition metals, which are able to doped ZnO nanoparticles. Such approach can help to improve the stability of ZnO nanoparticles in an eco-friendly manner by reducing the band gap. Titanium would not only be useful for controlling the unstable nature of nanoparticles, but also for enhancing their antimicrobial potential (Carofiglio et al., 2020).

Doped nanoparticles are typically characterized using various techniques including UV–visible spectroscopy, FT-IR, XRD, SEM, TEM, and Raman methods (Hamidian et al., 2021). XRD spectra show the phase purity of doped nanoparticles, FT-IR and UV–Visible spectra allow tracking of doping reaction and metal incorporation into the nanoparticle structure and morphological features are obtained through SEM and TEM [12]. The nature of microorganisms is evolving day by day due to severe environmental conditions and rates of survival, so there is a need to prepare an ultimate plan against these emerging pathogenic strains. The preparation of doped nanoparticles will be a promising way to combat these microorganisms due to their excellent antimicrobial and antioxidant properties [13]. Therefore, the aim of this study was to develop eco-friendly TiO_2_-doped ZnO nanocomposites and then well characterized in order to be used as antimicrobial agents against *Aspergillus niger* in the leather industry.

## 2 Materials and methods

The leather samples were randomly collected from the leather industry in Sialkot, Pakistan. The pure cultures of *Aspergillus niger, Escherichia coli*, and *Staphylococcus aureus* were isolated from collected leather samples. The black cardamom seeds were bought from the local market in Lahore, Pakistan.

### 2.1 Isolation and incubation of fungus

For fungal isolation, leather samples were placed inside a desiccator used as a tropical chamber source that was set at a temperature of 28 °C. The humidity required for fungal growth was maintained with autoclaved water to avoid bacterial contamination. Fungal growth was well observed after 7 days. The fungal samples were cultured on the Potato Dextrose Agar (PDA) with 1 mL of 10 % tartaric acid and 10 mg of streptomycin to avoid bacterial growth in the medium. Then 20 mL of media was poured into the plates, and fungal samples were incubated at 28 °C for two days. The isolated fungus was identified by microscope and characterized by Fungal Bank of University of the Punjab, Lahore, Pakistan.

### 2.2 Synthesis of TiO_2_ doped ZnO nanoparticles

First, Black cardamom seeds were carefully washed and dried at room temperature for 5 days. Then they were ground to a fine powder and only 5 mg of them were added to 100 mL distilled water. The mixture was stirred at 80 °C for 20 min, then filtered through filter paper (Whatman No. 1) and used as a reducing agent for the synthesis of titanium dioxide-doped zinc oxide nanoparticles ZnO/TiO_2_.

ZnO/TiO_2_ nanoparticles were prepared using the sol-gel method. 5 g of dihydrate zinc acetate (Zn (CH_3_COO)_2_∙2H_2_O) and 20 mL cardamom extract were added to 80 mL of distilled water and then stirred at 95 °C for 15 min. A solution of 2.5 g of titanium dioxide was prepared in 10 mL distilled water and then added to the previous ZnO solution. pH was adjusted to 7 with 1.5 N sodium hydroxide. The thick consistent paste obtained after constant stirring and heating for 14 h, was dried at 80 °C for 12 h. The resulting dried paste was then calcined at 500 °C for 2 h to obtain a fine nanoparticle powder.

### 2.3 Characterization of TiO_2_ doped ZnO nanoparticles

*UV–visible spectrophotometry:* The ZnO/TiO_2_ nanoparticles were dissolved in deionized water and UV–visible spectrum was obtained in the range of 200–400 nm by keeping deionized water as blank.

*Fourier transforms infrared spectroscopy (FT-IR):* The functional groups present in the ZnO/TiO_2_ nanoparticles were determined through FT-IR in dry air at room temperature. The potassium bromide pellet was employed to obtain the spectra from nanoparticles [14]. The IR analyzes were carried out using a Fourier transform infrared spectroscopy type ThermoFisher-Scientific Nicolet iS50. We proceeded with a resolution of 4 cm^−1^ and 16 accumulated scans. *FT-IR* was recorded in the range 400–4000 cm^−1^

*Scanning electron microscopy-energy dispersive X-ray analysis:* SEM and EDAX performed the morphology and elemental analysis of ZnO/TiO-2 nanoparticles, respectively. The SEM uses beam of electron instead of light to form significantly enlarged image of particles. Observations of surface morphology were performed using equipment Nova NanoSEM 450 analyzer. The results were observed by using the guidelines [15].

*X-ray diffraction:* The phase purity and crystalline structure of ZnO/TiO_2_ nanoparticles was examined through XRD. The structural study was performed by a diffractometer-Bruker AXS D8 Advance equipped with a copper anticathode (Cu K = 1.5402 A). Analysis of diffractograms was performed using a system based on data sheets ASTM (American Society for Testing and Materials) matching the interplanar spacing (d) 2θ recorded software. Conditions of the analyses were maintained constant for all samples in the interval 10°–90° in 2θ, for a step of 0.0101°/min.

### 2.4 Determination of antimicrobial activity of TiO_2_ doped ZnO nanoparticles

The agar well-diffusion method was applied to determine the antimicrobial nature of ZnO/TiO_2_ nanoparticles against gram-positive strain *Staphylococcus aureus,* gram-negative strain *Escherichia coli* and fungal specie *Aspergillus niger*. The nanoparticles were dissolved in deionized water to make final concentration 1 mg/mL. Potato-dextrose agar was used for fungal specie while LB agar was used for bacterial strains. A sterile cork borer was used to make three wells of 6 mm in each plate, and 100 μL of nanoparticles were added into the wells. The antibiotic Streptomycin was used as a positive control for bacterial samples plate and Fluconazole for fungal sample while normal saline was used as a negative control in each plate. The fungal plates were incubated at 28 °C for 48 h, while the bacterial samples were incubated at 37 °C for 24 h in aerobic conditions.

### 2.5 Determination of minimum inhibitory concentration (MIC) and biofilm inhibition

The standard broth dilution method was used to determine the antimicrobial efficacy of ZnO/TiO_2_ nanoparticles. Serial two-fold dilutions of nanoparticles were prepared in the range of 5–0.156 mg/mL. The microbial concentration was adjusted to 108 CFU/mL, 0.5 McFarland's standard, and used to determine the MIC in nutrient broth. The control sample contained inoculated broth, and the test samples were incubated at 37 °C for 24 h. The minimum inhibitory concentration was considered to be the value at which no visible growth was observed. The ability of ZnO/TiO_2_ nanoparticles for biofilm inhibition was determined by crystal violet (CV) dye assay. This method is based on the ability of CV dye binding to the extracellular polymeric substances and the bacterial cells. The total biofilm mass formation was quantified through this assay. The cultures of bacterial and fungal strains were prepared and incubated for 48 h at 37 °C. The nanoparticles were added in the test samples. Streptomycin and Fluconazole were used as a control of bacterial and fungal samples, respectively. The material of the test tubes was discarded after incubation, stained with 2 % crystal violet, gently washed with phosphate buffer saline (PBS), and dissolved in 30 % glacial acetic acid. To determine anti-biofilm activity of nanoparticles, the absorbance was measured at 570 nm.

## 3 Results and discussion

### 3.1 Microscopic analysis of isolated fungus

The microscopic examination of isolated fungal samples indicated the presence of *Aspergillus niger*. In addition, it showed tiny, upright conidiophores, phialides growing from the centre of spore and small globule swelling at the ends. The conidia were light to dark brown, single celled and globule shaped.

### 3.2 TiO_2_ doped ZnO nanoparticles

The fine powder of TiO_2_ doped ZnO nanoparticles were obtained by the chemical reaction of titanium dioxide and zinc acetate dihydrate, under high temperature and pressure. The quality of nanoparticles was analyzed by observing the color changed from yellow to white during this chemical reaction. The powder was collected from the bottom of beaker, calcined and then characterized.

### 3.3 Characterization of ZnO/TiO_2_ NPs

#### 3.3.1 UV–Visible spectrophotometric analysis

**Fig. 2:**
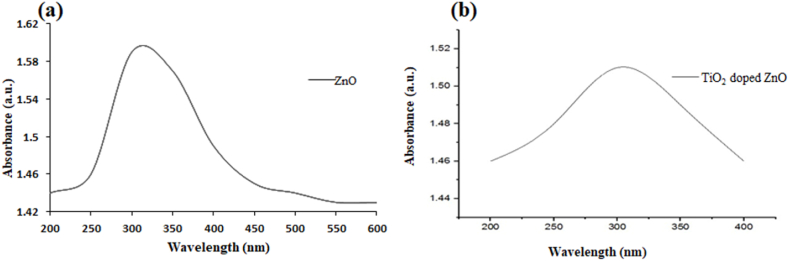
UV–Visible spectra of (a) ZnO nanoparticles and (b) titanium dioxide doped ZnO NPs.

The optical properties of ZnO and ZnO/TiO_2_ NPs were analyzed by UV spectroscopy in the wavelength range 200–400 nm. The UV–Visible spectrum, shown in Fig. 2, showed an absorbance maximum from ZnO/TiO_2_ NPs between 300 and 350 nm. Before doping with TiO_2,_ the maximum peak for ZnO nanoparticles was obtained at 300 nm as shown in Fig. 2a while after doping, the peak shifted between to 320 nm as shown in Fig. 2b. Moreover, after doping with TiO_2_, the peak absorbance was decreased and peak edge shifted to higher wavelength. This is due to the conformational changes happened in the crystal lattice of ZnO nanoparticles. The insertion of Ti ions from TiO_2_ into the crystal lattice of ZnO nanoparticles disturbed its optical properties and revealed different absorption spectra. After successful doping with TiO_2_, the resultant product exhibited more visible light absorbance as compared to ZnO nanoparticles; this enhancement is likely due to the fact that the light scattering ability of ZnO nanoparticles was improved due to the incorporation of titanium ions into the crystal lattice [16]. Samuel et al., 2022, who obtained an absorbance maximum of ZnO/Ti nanoparticles at 365 nm, not far from that obtain this result. In their research, zinc nitrate and hydrazine hydrate were used as precursors for the synthesis of these nanoparticles (Samuel et al., 2022). Another study was conducted using zinc acetate dihydrate as a precursor through Sol gel technique but with different doping element and capping agent and showed a maximum of absorbance around 397 nm [17].

#### 3.3.2 Fourier transforms infrared spectroscopy (FT-IR)

**Fig. 3:**
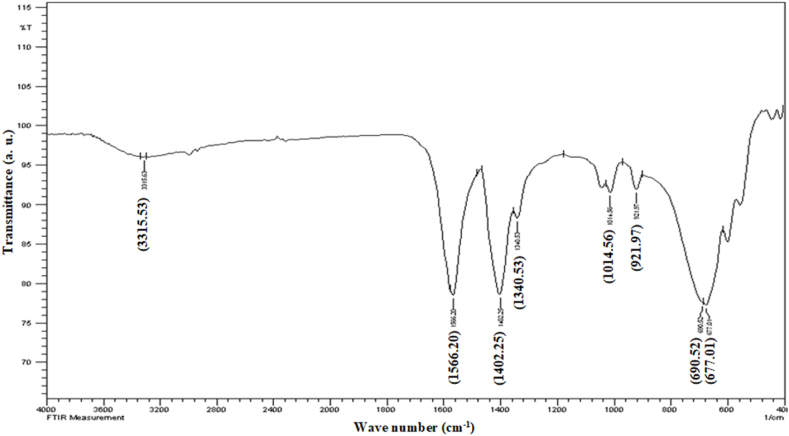
FT-IR spectrum of ZnO/TiO_2_ NPs.

The various chemical bonds, especially those formed during nanoparticle synthesis, were studied by FT-IR spectroscopy. The insertion of a transition metal (titanium) into the nanoparticle crystal lattice disrupted the structure of zinc oxide crystals. The FT-IR spectrum was obtained in the range of 4000 to 400 cm^−1^ and different bands were observed at 3315.53, 1566.20, 1402.25, 1340.53, 1014.56, 921.97, 690.52, and 677.01 cm^−1^ (Fig. 3). A weak absorption band was observed at 3443 cm^−1^ which indicated the hydroxyl group stretching modes [18]. The absorbance bands observed in the range of 1100 cm^−1^ to 1600 cm^−1^ showed the presence of -OH bending vibrational modes, -C-OH bending and stretching in its own plane and out of the plane [19]. The small bands between 800 cm^−1^ and 1050 cm^−1^ showed the asymmetric-symmetric stretching modes of zinc oxide and zinc carboxylates [20]. Some broad bands were also observed at 690 cm^−1^ and 677.01 cm^−1^ which represents the vibrational modes of zinc oxide bonds [19].

Wongrerkdee et al. prepared titanium doped ZnO nanoparticles by the reaction of zinc nitrate hexahydrate with ethoxyethanol (C_4_H_10_O_2_) through rapid combustion technique. Their FT-IR spectra showed similar absorption bands, although they prepared the nanoparticles with different precursors [21].

#### 3.3.3 Scanning electron microscopy-energy dispersive X-ray (SEM-EDAX)

**Fig. 4:**
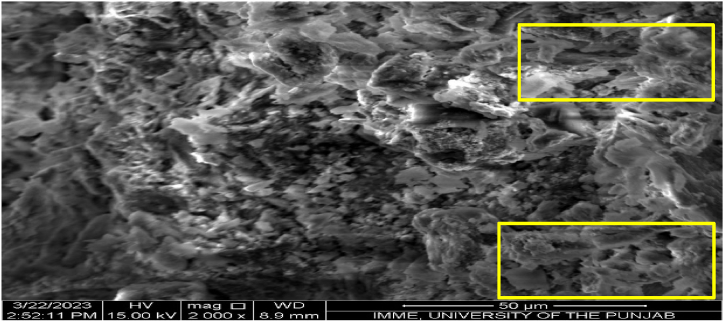
Representative image of scanning electron microscopy (SEM) showing the morphological feature of ZnO/TiO_2_ NPs.

The high resolution surface morphology and characteristics of ZnO/TiO_2_ NPs were evaluated through scanning electron microscopy (SEM). In scanning electron microscopy, interaction of electrons with the surface of materials produced a three dimensional structure. Fig. 4 shows the roughly spherical and agglomerated structure of nanoparticles under 2000× magnification at 50 μm. With a uniform distribution of particles, ZnO/TiO_2_ averaging 45.44 nm in size. Vishwakarma & Singh synthesized ZnO nanoparticles by using zinc acetate dihydrate through Sol Gel technique and their SEM analysis have reported spherical shaped nanoparticles in the size range of 15–25 nm [22]. Similarly, colloidal nano spheres of ZnO nanoparticles were prepared with zinc nitrate and ammonium hydroxide using controlled precipitation method [18]. Ullah et al. reported that nanoparticle size varied with the pH of the reaction medium, they increased with pH and even changed color.

**Fig. 5:**
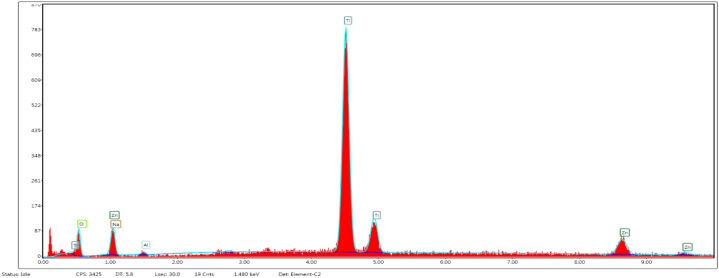
Representative image of Energy Dispersive X-ray analysis of ZnO/TiO_2_ NPs.

In the present work, the scanning electron microscope (SEM) was equipped with Energy Dispersive X-ray (EDAX) model which provided elemental analysis of ZnO/TiO_2_ NPs as shown in Fig. 5. The result showed the Ti-rich profiles with the presence of Zn, Al, Na and oxygen.

#### 3.3.4 X-ray diffraction (XRD) analysis

**Fig. 6:**
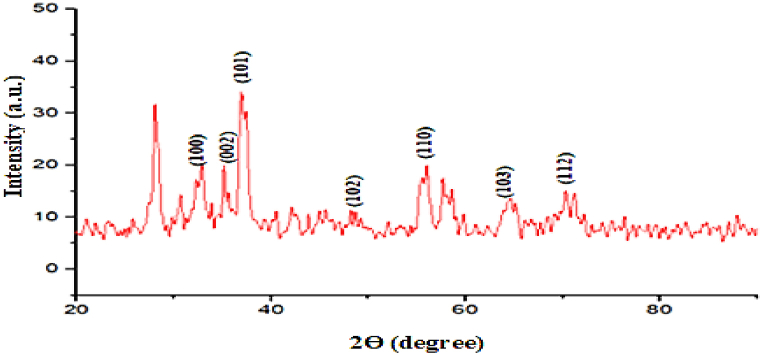
X-Ray diffraction (XRD) pattern of titanium doped ZnO nanoparticles.

To determine the crystal structure of TiO_2_ doped ZnO nanoparticles, the X-ray diffraction (XRD) was applied on ZnO/TiO_2_ NPs. Fig. 6 showed well-defined peaks at 2Ө of 32°, 35.5°, 37.2°, 55.6° which intimated to the crystal planes of (100), (002), (101), (110) respectively, while some weak and broad peaks were determined at 2Ө of 47.9°, 63.51°, and 70° which leads to the crystal planes of (102), (103), and (112) respectively. All these pattern peaks coincided with the reported data of ZnO/TiO_2_ NPs [21]. Three major peaks, as shown in Table 1, of (100), (002) and (101) were considered important for crystalline size measurement of titanium doped ZnO nanoparticles. However, the peaks below 30° may be linked to the orthorhombic phases of Zn(OH)_2_. Ramesh Ade et al., 2021 have performed similar study. They have investigated the impact of titanium ions doping on the structural, optical, morphological, and photoluminescence properties of ZnO nanoparticles. Their XRD spectrum showed peaks which are concurred with the peak pattern of our research. These findings provide clear evidence for the correct formation of ZnO/TiO_2_ nanoparticles in the current study.

**Table 1:** XRD pattern peak for titanium dioxide doped ZnO nanoparticles.

**Peak No.**	**Angle 2Ө**	**Planes**
1.	32°	100
2.	35.5°	002
3.	37.2°	101
4.	47.9°	102
5.	55.6°	110
6.	63.51°	103
7.	70°	112

### 3.4 Antimicrobial activity of ZnO/TiO_2_ NPs

**Fig. 7:**
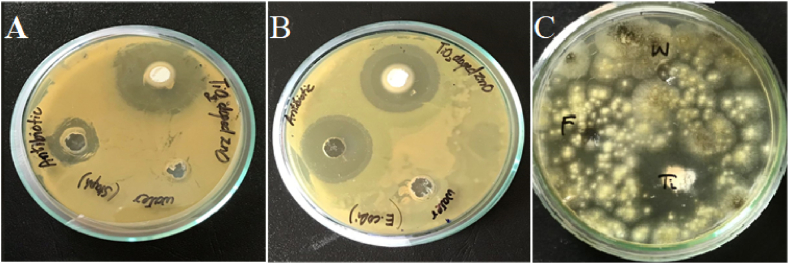
Zones of inhibition of ZnO/TiO_2_ nanoparticles showing antimicrobial potential against (A) gram-positive strain *Staphyloccus aureurs*, (B) gram-negative strain *Escherichia coli*, (C) fungal strain *Aspergillus niger* through agar well diffusion method.

The titanium dioxide doped ZnO nanoparticles were tested for their antimicrobial potential through agar well diffusion method. Three different strains of microorganisms including bacteria and fungi were selected for experimental protocol. The synthesized nanoparticles showed potent antimicrobial action against all selected microorganisms. Fig. 7 (A, B, C) have proven clear zones of inhibition against microorganisms due to the high bacterial membrane degradation rate of ZnO nanoparticles. The maximum zone of inhibition due to ZnO/TiO_2_ nanoparticles was observed on the plates of *Aspergillus niger* 30.16 ± 1.20 mm (Fig. 7C) followed by *Escherichia coli* 30 ± 0.77 mm (Fig. 7B) and *Staphylococcus aureus* 29 ± 0.37 mm (Fig. 7A). The recorded diameter of clear zones of inhibitions is also showed in Table 2. These results have verified the role of ZnO/TiO_2_ nanoparticles against microorganism especially against fungal strains [23]. has prepared chromium doped ZnO nanoparticles by using same protocol as described in this study. They have tested the antimicrobial potential of these nanoparticles against different bacteria strains and fungal strains. The maximum zone of inhibition was shown against *K. pneumoniae* 36 mm while least zone of inhibition observed against *C. tropicalis* 14 mm. Their results differ slightly due to the use of different bacterial and fungal strains in both studies. Each microorganism shows diversity in drug resistance nature, which can change the antimicrobial potential of nanoparticles against these pathogens. Another study has synthesized ZnO nanoparticles *via* two routes i.e. by chemical approach and green synthesis in garlic extracts. They depicted positive results about the antifungal activity of ZnO nanoparticles, as their nanobiohybrids from green synthesis exhibited 72 % fungal growth inhibition and 87 % inhibition through chemical approach [24]. The antifungal potential of ZnO nanoparticles, synthesized by using ethylene glycol and acetic acid as solvents, was investigated against *Aspergillus niger and Botrytis cinerea*. Due to the presence of different solvents, change in morphology of nanoparticles was observed which also influenced the antifungal activity, as acicular nanoparticles obtained from acetic acid showed enhanced antifungal activity rather than spheroidal nanoparticles obtained from ethylene glycol [25]. These results are the clear evidence for antimicrobial activity of ZnO nanoparticles.

**Table 2:** Antimicrobial potential of ZnO/TiO_2_ nanoparticles against *Aspergillus niger, Staphylococcus aureus* and *Escherichia coli.*

**Samples**	**Diameter of zones of inhibition (mm)**		
	***Aspergillus niger***	***Staphylococcus aureus***	***Escherichia coli***
ZnO/TiO_2_ NPs	30.16 ± 1.20^a^	29 ± 0.37^a^	30 ± 0.77^a^
Streptomycin	–	30 ± 0.55^a^	30.9 ± 0.81^a^
Fluconazole	25.9 ± 0.33^a^	–	–
Normal saline	–	–	–

All the values are expressed as mean ± standard deviation. Streptomycin = Positive control for bacterial strains, Fluconazole = Positive control for fungal strain, Normal Saline = Negative control, and no activity was represented by (−).

a No significant difference between these values according to one way ANOVA, (F_2,6_ = 0.00 P = 0.998).

### 3.5 Minimum inhibitory concentration (MIC) of TiO_2_ doped ZnO nanoparticles

Due to the antimicrobial potential of ZnO/TiO_2_ nanoparticles, their minimum inhibitory concentration (MIC) was determined through a micro-dilution assay, with dilutions ranging from 5 to 0.156 mg mL^−1^. Table 3 demonstrates the MIC values of ZnO/TiO_2_ nanoparticles against selected microorganisms. For *Aspergillus niger,* a dilution of 0.625 mg/mL of nanoparticles was the MIC value, while for bacterial strains, it was 0.312 mg mL^−1^. The minimum the value of MIC more it will be effective against pathogens. The results have shown that the MIC values of nanoparticles were less for bacterial strains rather than fungal strains, which proved that these bacterial strains are more susceptible to ZnO/TiO_2_ nanoparticles [26]. used green synthesis approach to prepared copper doped ZnO nanoparticles and characterized through different techniques. The MIC values of these nanoparticles were determined through two-fold serial dilution (SLD) methods with the dilutions ranges from 20 to 1.25 μg/mL. Their study showed positive result with least MIC values which indicated the excellent antimicrobial potential of prepared nanoparticles. The variation in the values of MIC may be due to different doping material and different precursor compounds for nanoparticles. The doping material can influence the killing of pathogens through nanoparticles.

**Table 3:** Minimum Inhibitory concentration (MIC) in mg/mL of ZnO/TiO_2_ nanoparticles for *Aspergillus niger, Staphylococcus aureus* and *Escherichia coli*.

**Microorganisms**	**Minimum Inhibitory Concentrations (MIC)**			
	**(mg/mL)**			
	**ZnO/TiO**_**2**_	**Streptomycin**	**Fluconazole**	**Normal Saline**
***Aspergillus niger***	0.625	–	1.25	–
***Staphylococcus aureus***	0.312	0.15	–	–
***Escherichia coli***	0.312	0.15	–	–

Streptomycin = Positive control for bacterial strains, Fluconazole = Positive control for fungal strains, Normal Saline = Negative control.

The ZnO nanoparticles were synthesized through Sol-gel technique with different reaction times in order to prepare variable sized nanoparticles. The size and surfaces of nanoparticles were controlled to evaluate their antimicrobial efficacy against *Staphylococcus aureus* and *Escherichia coli*. The results showed increased MIC with increased reaction time [27]. Similarly, the antimicrobial activity and minimum inhibitory concentration of ZnO, TiO_2_ and silver nanoparticles were determined against various pathogenic strains i.e. *Salmonella typhimurium, Brucella abortus,* and *Candida albicans*. Their research findings strongly suggested the antibacterial and antifungal potential of all selected nanoparticles [28].

### 3.6 Anti-biofilm inhibition analysis of TiO_2_ doped ZnO nanoparticles

The biofilm production is one of the microbial properties, which exhibit through complex polysaccharides from group of microbial strains for their growth and survival. The prepared ZnO/TiO_2_ nanoparticles were investigated for their anti-biofilm potential against these pathogenic strains. The optical density (OD) of each sample was examined to ascertain the biofilm formation. Table 4 shows different ODs in mean ± SD for biofilm production in the samples of *Aspergillus niger, Staphylococcus aureus* and *Escherichia coli* which do not contain solution of nanoparticles while ZnO/TiO_2_ containing samples showed no density in each microbial strain. These results clearly proved the excellent antibacterial and antifungal nature of ZnO/TiO_2_ nanoparticles. A study was performed by Ref. [29] through biogenic synthesis of ZnO nanoparticles by using aqueous extract of dried powder of *Emblica officinalis* (Amla). In results, the prepared nanoparticles were capable to inhibit bacterial adhesion and corresponding density in the biofilms. Subsequently, another experiment was performed to determine the anti-biofilm potential of ZnO nanoparticles against various resistive microorganisms. Their outcomes have supported our research findings, endorsing the accuracy of our results [30]. Hussain Udayagiri et al., 2024 has synthesized ZnO nanoparticles through bio-reduction of aqueous zinc nitrate solution with the help of various phytochemicals from *Echinochloacolona* leaf extract and investigated the biofilm inhibition mechanism of prepared nanoparticles. The ZnO nanoparticles exhibited significant anti-biofilm potential against pathogenic microorganisms which proposing that these nanoparticles have excellent antimicrobial efficacy [31]. All these results showed that our findings are consistent with previously published studies which further validate our research.

**Table 4:** Optical density (OD) showing anti-biofilm potential of ZnO/TiO_2_ nanoparticles against *Aspergillus niger, Staphylococcus aureus* and *Escherichia coli*. Culture broth showed OD = 0.16 ± 0.2.

**Samples**	**Optical Density (OD)**		
	***Aspergillus niger***	***Staphylococcus aureus***	***Escherichia coli***
**ZnO/TiO**_**2**_	–	–	–
**Non ZnO/TiO**_**2**_	1.87 ± 0.12^a^	1.66 ± 0.12^a^	1.84 ± 0.07^a^
**Streptomycin**	–	–	–
**Fluconazole**	–	–	–

a No significant difference between the triplicates according to one way ANOVA.

## 4 Conclusion

This study has provided a safe and environmental-friendly approach to synthesize titanium dioxide-doped ZnO nanoparticles. Black cardamom seed's extract was used as the reducing agent for these nanoparticles. The synthesized nanocomposite was characterized through UV–visible spectrophotometry, FT-IR, SEM-EDAX, and XRD. The obtained results have confirmed the accurate synthesis of ZnO/TiO_2_ nanoparticles. These nanoparticles are prepared without impurities and their antimicrobial potential is evaluated against different fungal and bacterial strains. Results showed outstanding antimicrobial activity against all the strains with acceptable MIC and anti-biofilm potential. Enhanced antimicrobial potential were obtained due to the doping compound (TiO_2_) which incorporated into the crustal lattice of ZnO nanoparticles. The reason of tremendous microbial damage is due to the insertion of Zn^2+^ ions into the interior of microbial cell through electrostatic force of attraction, damages the DNA and cellular organelles of microorganisms.

## Funding

No Funding available for this work.

## Data availability statement

All the data used in this study are already present in this manuscript.

## Ethical concern

No animal or plant were used in this study, so no ethical concern was raised during this work.

## Research involving cell lines

Not Applicable.

## Research involving plants

No self-cultivated or wild plant was used so not Applicable.

## CRediT authorship contribution statement

**Asma Irshad:** Writing – review & editing, Project administration, Conceptualization. **Rabbia Jawad:** Validation, Software, Resources. **Uzair Ishtiaq:** Writing – original draft, Validation, Resources, Project administration, Investigation, Funding acquisition, Formal analysis, Conceptualization. **Nicolas Joly:** Validation, Methodology, Formal analysis. **Bochra Bejaoui:** Visualization, Methodology, Data curation. **Naceur M'Hamdi:** Methodology, Investigation. **Patrick Martin:** Writing – review & editing, Visualization, Validation, Supervision, Resources, Project administration, Investigation, Funding acquisition, Conceptualization. **Firdous Mubashar:** Formal analysis.

## Declaration of competing interest

The authors declare that they have no known competing financial interests or personal relationships that could have appeared to influence the work reported in this paper.

## References

[1] Roh E.K. (2020). Mechanical properties and preferences of natural and artificial leathers, and their classification with a focus on leather for bags. Journal of Engineered Fibers and Fabrics.

[2] Abdel-Maksoud G., Abdel-Nasser M., Sultan M.H., Eid A.M., Alotaibi S.H., Hassan S.E.-D., Fouda A. (2022). Fungal biodeterioration of a historical manuscript dating Back to the 14th century: an insight into various fungal strains and their enzymatic activities. Life.

[3] Nyakundi J.O. (2023). Bacterial and fungal damage in leather. J. Am. Leather Chem. Assoc..

[4] Khambhaty Y., Samidurai S. (2024). An insight into the microbiome associated with the damage of raw animal hide and skin-primarily protein, during leather making. Int. J. Biol. Macromol..

[5] Rossi M., Papetti A., Marconi M., Germani M. (2021). Life cycle assessment of a leather shoe supply chain. Int. J. Sustain. Eng..

[6] Thakur R., Yadav S. (2024). Smart multifaceted potential microbial inoculant isolated from rhizospheric soils of Bergenia ciliata and possible role in developing green biosynthesized nanoparticles. Biocatal. Agric. Biotechnol..

[7] Mehtab A., Ahmed J., Alshehri S.M., Mao Y., Ahmad T. (2022). Rare earth doped metal oxide nanoparticles for photocatalysis: a perspective. Nanotechnology.

[8] Chandra H., Kumari P., Yadav A., Yadav S. (2022). Green Nanomaterials for Industrial Applications.

[9] Čuk N., Šala M., Gorjanc M. (2021). Development of antibacterial and UV protective cotton fabrics using plant food waste and alien invasive plant extracts as reducing agents for the in-situ synthesis of silver nanoparticles. Cellulose.

[10] Vinotha V., Yazhiniprabha M., Raj D.S., Mahboob S., Al-Ghanim K.A., Al-Misned F., Govindarajan M., Vaseeharan B. (2020). Biogenic synthesis of aromatic cardamom-wrapped zinc oxide nanoparticles and their potential antibacterial and mosquito larvicidal activity: an effective eco-friendly approach. J. Environ. Chem. Eng..

[11] Muktaridha O., Adlim M., Suhendrayatna S., Ismail I. (2021). Progress of 3d metal-doped zinc oxide nanoparticles and the photocatalytic properties. Arab. J. Chem..

[12] Alaizeri Z.M., Alhadlaq H.A., Aldawood S., Akhtar M.J., Amer M.S., Ahamed M. (2021). Facile synthesis, characterization, photocatalytic activity, and cytotoxicity of ag-doped mgo nanoparticles. Nanomaterials.

[13] Nigam A., Pawar S. (2020). Structural, magnetic, and antimicrobial properties of zinc doped magnesium ferrite for drug delivery applications. Ceram. Int..

[14] Irshad A., Sarwar N., Sadia H., Riaz M., Sharif S., Shahid M., Khan J.A. (2020). Silver nano-particles: synthesis and characterization by using glucans extracted from Pleurotus ostreatus. Appl. Nanosci..

[15] Samy A., El-Sherbiny A.E., Menazea A. (2019). Green synthesis of high impact zinc oxide nanoparticles. Egypt. J. Chem..

[16] Rahman M.U., Wei M., Xie F., Khan M. (2019). Efficient dye-sensitized solar cells composed of nanostructural ZnO doped with Ti. Catalysts.

[17] Rajendran R., Mani A. (2020). Photocatalytic, antibacterial and anticancer activity of silver-doped zinc oxide nanoparticles. J. Saudi Chem. Soc..

[18] Ullah S., Gulnaz A., Anwar S., Kamal A., Wali H. (2024). Synthetization and characterization of zinc oxide nanoparticles by X-ray diffractometry (XRD), fourier transforms, infra-red spectroscopy (FT-IR), scanning electron microscopy (SEM) and antibacterial activity test. American Journal of Physical Sciences.

[19] Darmadi I., Taufik A., Saleh R. (2020). Paper Presented at the Journal of Physics: Conference Series.

[20] Menazea A., Awwad N.S. (2020). Antibacterial activity of TiO2 doped ZnO composite synthesized via laser ablation route for antimicrobial application. J. Mater. Res. Technol..

[21] Wongrerkdee S., Wongrerkdee S., Boonruang C., Sujinnapram S. (2022). Enhanced photocatalytic degradation of methylene blue using Ti-doped ZnO nanoparticles synthesized by rapid combustion. Toxics.

[22] Vishwakarma A., Singh S.P. (2020). Synthesis of zinc oxide nanoparticle by sol-gel method and study its characterization. Int. J. Res. Appl. Sci. Eng. Technol..

[23] Rajivgandhi G.N., Ramachandran G., Alharbi N.S., Kadaikunnan S., Khaleed J.M., Manokaran N., Li W.-J. (2021). Substantial effect of Cr doping on the antimicrobial activity of ZnO nanoparticles prepared by ultrasonication process. Mater. Sci. Eng., B.

[24] Patino-Portela M.C., Arciniegas-Grijalba P.A., Mosquera-Sanchez L.P., Sierra B.E.G., Munoz-Florez J.E., Erazo-Castillo L.A., Rodriguez-Paez J.E. (2021). Effect of method of synthesis on antifungal ability of ZnO nanoparticles: chemical route vs green route. Advances in nano research.

[25] Erazo A., Mosquera S.A., Rodríguez-Paéz J. (2019). Synthesis of ZnO nanoparticles with different morphology: study of their antifungal effect on strains of Aspergillus Niger and Botrytis cinerea. Mater. Chem. Phys..

[26] Okeke I., Agwu K., Ubachukwu A., Maaza M., Ezema F. (2020). Impact of Cu doping on ZnO nanoparticles phyto-chemically synthesized for improved antibacterial and photocatalytic activities. J. Nanoparticle Res..

[27] da Silva, B. L., Caetano, B. L., Chiari-Andréo, B. G., Pietro, R. C. L. R., & Chiavacci, L. A. (2019). Increased antibacterial activity of ZnO nanoparticles: Influence of size and surface modification. Colloids Surf. B Biointerfaces, 177, 440-447. doi:10.1016/j.colsurfb.2019.02.013. 30798065

[28] Mohammed A.K., Salh K.K., Ali F.A. (2021). ZnO, TiO2 and Ag nanoparticles impact against some species of pathogenic bacteria and yeast. Cell. Mol. Biol..

[29] Kaur T., Putatunda C., Vyas A., Kumar G. (2021). Zinc oxide nanoparticles inhibit bacterial biofilm formation via altering cell membrane permeability. Prep. Biochem. Biotechnol..

[30] Lahiri D., Ray R.R., Sarkar T., Upadhye V.J., Ghosh S., Pandit S., Pati S., Edinur H.A., Abdul Kari Z., Nag M. (2022). Anti-biofilm efficacy of green-synthesized ZnO nanoparticles on oral biofilm: in vitro and in silico study. Front. Microbiol..

[31] Udayagiri H., Sana S.S., Dogiparthi L.K., Vadde R., Varma R.S., Koduru J.R., Ghodake G.S., Somala A.R., Boya V.K.N., Kim S.-C. (2024). Phytochemical fabrication of ZnO nanoparticles and their antibacterial and anti-biofilm activity. Sci. Rep..

